# Baroreflex Activation Therapy for the Treatment of Drug-Resistant Hypertension: New Developments

**DOI:** 10.1155/2012/587194

**Published:** 2012-06-12

**Authors:** Teba Alnima, Peter W. de Leeuw, Abraham A. Kroon

**Affiliations:** Department of Internal Medicine, Maastricht University Medical Center, P. Debyelaan 25, 6229 HX Maastricht, The Netherlands

## Abstract

In the past few years, novel accomplishments have been obtained in carotid baroreflex activation therapy (BAT) for the treatment of resistant hypertension. In addition, this field is still evolving with promising results in the reduction of blood pressure and heart rate. This overview addresses the latest developments in BAT for the treatment of drug-resistant hypertension. Although not totally understood considering the working mechanisms of BAT, it appeared to be possible to achieve at least as much efficacy of single-sided as bilateral stimulation. Therefore unlike the first-generation Rheos system, the second-generation Barostim *neo* operates by unilateral baroreflex activation, using a completely different carotid electrode. Also significant improvements in several cardiac parameters have been shown by BAT in hypertensive patients, which set the basis for further research to evaluate BAT as a therapy for systolic heart failure. Yet important uncertainties need to be clarified to guarantee beneficial effects; hence not all participants seem to respond to BAT.

## 1. Introduction

The urgent need for treatments for drug-resistant hypertension has engendered great interest in the development of new approaches. Carotid baroreflex activation is a relatively novel therapy for drug-resistant hypertension. Several trials have demonstrated the safety and efficacy of this method in patients with treatment-resistant hypertension [1, 2]. In addition, the benefits of baroreflex activation therapy (BAT) appear to extend beyond blood pressure (BP) reduction and may be applicable for related cardiovascular disorders.

Recently, several reviews have been published on the role of baroreceptors in BP regulation, the history of BAT as a strategy to correct high BP, and the accomplishments of the carotid baropacer Rheos system. Therefore, this paper will focus primarily on the latest human developments in the field of BAT for therapy-resistant hypertension. Prevailing theories and hypotheses that have led to these new developments will also be discussed. In addition, we will evaluate the potential indication of BAT in the treatment of heart failure.

## 2. Carotid Baroreceptors and Mechanisms of BAT

It is widely known that the baroreceptors are mechanosensory nerve endings situated in the inner adventitia of the arterial wall of carotid sinus and aortic arch and are mainly associated with BP regulation [3, 4]. Functional anatomy and neurophysiology of carotid baroreflex system have been extensively described in several reviews and for further explanation we refer to cited articles [5–7]. 

### 2.1. Electrical Carotid Baroreceptor or Baroreflex Activation?

During our literature search we noticed two main denominations of BAT carotid baroreceptor stimulation and carotid baroreflex stimulation. Do we know what is stimulated by a baropacer: receptor or fibre? 

When a mechanosensory receptor is stretched, depolarization may arise by deformation of ionic pores, change in chemical compounds inside the cell as a result of stretching, and a transient rise in the membrane potential while the membrane is being stretched [8]. Normally, an increase in arterial BP is sensed by baroreceptors through their mechanical deformation during vascular stretch. Evidence suggests that sodium and calcium influx through mechanosensitive ion channels is responsible for depolarization of baroreceptors during vessel wall deformation [9]. The ion channel ASIC2 (least acid sensitive subunit), member of the DEG/ENaC family, appears to be an important determinant of the arterial baroreceptor complex [10]. Subsequently, signals from the baroreceptors need to be transformed into action potentials in order to be transported to the central nervous system (CNS). This mechanoelectrical transformation occurs in the spike-initiating zone (SIZ) near the nerve terminals [8, 11]. When depolarization reaches a specific threshold, voltage-dependent sodium and potassium channels are opened to generate action potentials [9]. These action potentials are then transported through nerve fibres to CNS for further processing.

Adaptation and resetting are important and remarkable characteristics of baroreflexes [12, 13]. Baroreceptor activity is not maintained during sustained mechanical changes. The activity of baroreceptors increases only initially when the receptors are stimulated but declines over time (adaptation). In addition, the pressure threshold of baroreceptor activation is increased after a period of acute BP increase (resetting). The mechanisms of resetting are not totally clarified yet, but a number of factors including vascular wall distensibility, alteration in the coupling between receptors and vascular wall, and receptor properties have been proposed to play a role in this process [14, 15]. 

Although the exact mechanisms of mechanosensation of the baroreceptor are still not totally clear yet, we think that the current baropacers probably do not stimulate the mechanosensor itself, as it is mainly activated by its specific stimulus (stretch). However, electrical stimulation theoretically may cause ion channels of all types to change their conformation. Moreover, there are several known substances which are able to modulate the sensitivity of the baroreceptor response (e.g., angiotensin II, aldosterone) [16]. In our opinion induction of an electrical charge to the carotid sinus wall by a baropacer is more likely to have an effect in the SIZ, which is rich in voltage-gated channels, and the nerve fibres. These areas are more sensitive to electrical charge, which can result in the generation of action potentials. Another argument which supports the idea of mechanotransduction bypass during BAT is the absence of adaptation and resetting in long-term BAT. The BP decrease in subjects treated with BAT is sustained, even after years of continuous therapy with the device [17]. In addition, we observed in a limited number of patients an increase in BP to pretreatment levels after cessation of long-term BAT, which is consistent with existing animal data [18]. In case baroreceptors will indeed be activated by continuous BAT, they will probably undergo desensitization and response adaptation and theoretically reset to the prevailing BP decrease even after turning off the device. For these reasons it is more likely to assume that the SIZ and nerve fibres are activated than the receptor itself during electrical baropacing. Anyhow, the stated arguments are all indirect evidence for either baroreflex or baroreceptor activation and require further research. Therefore, we suggest that the term baroreflex activation is preferable, as it indicates the general mechanism of BAT.

### 2.2. Working Mechanisms of BAT

Although several studies provided evidence of persistent BP and heart rate reduction by BAT, the exact neural mechanisms underlying these effects remain to be determined. Two studies revealed a part of the working mechanisms of the baropacer. Heusser et al. reported in a study with 12 patients an acute and sharp decrease in muscle sympathetic nerve activity (MSNA) when electric carotid baroreflex activation was started [19]. This observation was associated with a significant systolic BP decrease from 193 ± 9 mmHg to 161 ± 10 mmHg, which can be explained by a decrease in sympathetic vasomotor tone. In another study, Wustmann et al. analyzed heart rate variability and heart rate turbulence in 21 patients using 24-hour ECG before device activation and 3 months after device activation. This study showed, next to a significant BP decrease from 185 ± 31/109 ± 24 mmHg to 154 ± 23/95 ± 16 mmHg, sustained changes in heart rate variability and heart rate turbulence [20]. Despite their small sample size, these studies suggest that BAT induces a decrease in sympathetic activity and an increase in parasympathetic activity. 

The aforementioned studies also indicate the potential role of baroreceptors in long-term BP regulation. The baroreceptors have been known for a long time to be mainly responsible for short-term BP regulation. However, their function in long-term control of BP has been repeatedly argued. The main arguments against baroreceptor involvement in long-term BP regulation were the little effect on the average mean arterial pressure (MAP) after sinoaortic denervation and baroreceptor resetting towards imposed BP changes [21–23]. On the other hand, investigators including Sleight and Thrasher have presented results that favor the idea of a role for baroreceptors in longer term BP levels [24, 25]. In addition, long-term results of BAT in treatment-resistant hypertension are consistent with this idea. Several human studies (DEBuT-HT and Pivotal Trial) demonstrated that prolonged activation of the carotid baroreflex has the capability of producing significant and sustained reductions in BP without any trend for adaptation. In a single-center study, BAT showed a pronounced BP decrease of even 53/30 mmHg after 4 years of continuous therapy in subjects with drug-resistant hypertension [17]. This clearly suggests a potential role for baroreflexes in long-term control of arterial pressure. 

Nevertheless, not all patients implanted with a baropacer showed a response to BAT. Recent long-term Pivotal data demonstrated a clinically significant response to BAT in 88% of participants, in which a response was defined as achievement of goal systolic BP (≤140 mmHg or ≤130 mmHg in diabetes or renal disease) or a drop in systolic BP ≥20 mmHg from start of therapy [26]. In addition, a great variability in response has been observed. This may be attributed to various subject characteristics, for instance, carotid sinus anatomy, race, weight, comorbidity, accuracy of surgical implantation, concomitant medication use, and so forth. Moreover, the contribution of different genetic and metabolic factors in the pathophysiology of (resistant) hypertension may also play a role in the response to BAT. Therefore, future studies need to focus on adequate patient selection for BAT. 

## 3. Novel Carotid Baroreflex Activation Devices

Electrical activation of the carotid baroreflex in resistant hypertension is not a new concept. In the past several investigators reported on carotid baroreflex activation in patients with resistant hypertension and angina pectoris. For a summary of the history of BAT we refer to the review of Scheffers et al. [27]. Although previous experiences with carotid baropacing reported a consistent BP drop in the majority of participants, the use of carotid baroreflex activation remained very restricted to experimental settings. The main reasons for the therapy not achieving common clinical usage were the technical and surgical limitations at that time [28]. 

CVRx Inc. (Minneapolis, MN, USA) has developed a novel approach for implantable carotid baroreflex activation systems. The first generation Rheos system consists of an implantable pulse generator (IPG) and two carotid sinus electrodes, which were bilaterally implanted by a surgical procedure [29]. Device description and implantation procedure have been explained in detail by Tordoir et al. [30]. 

**Figure 1:**
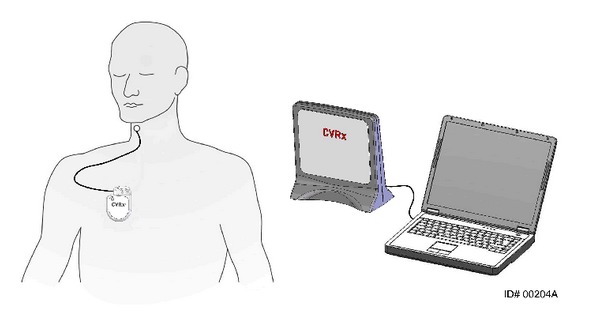
Barostim *neo* system consisting of an implantable pulse generator and a unilateral electrode. Programming is performed by a computer system, which connects to the device by wireless telemetry. Figure permission was granted by CVRx, Inc.

**Figure 2:**
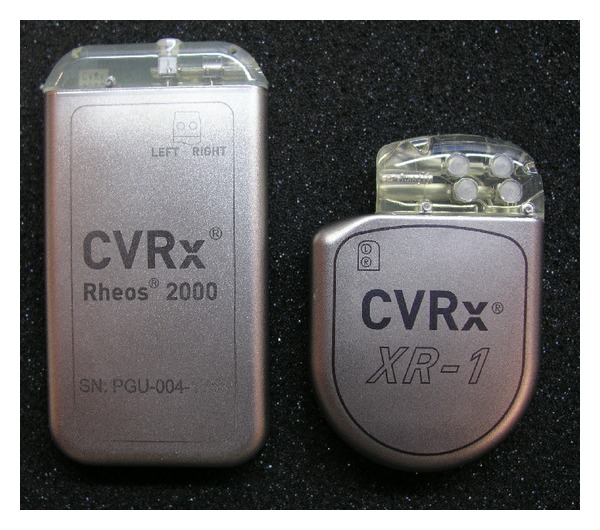
The implantable pulse generators of the Rheos system (on the left) and Barostim *neo* (on the right). Figure permission was granted by CVRx, Inc.

**Figure 3:**
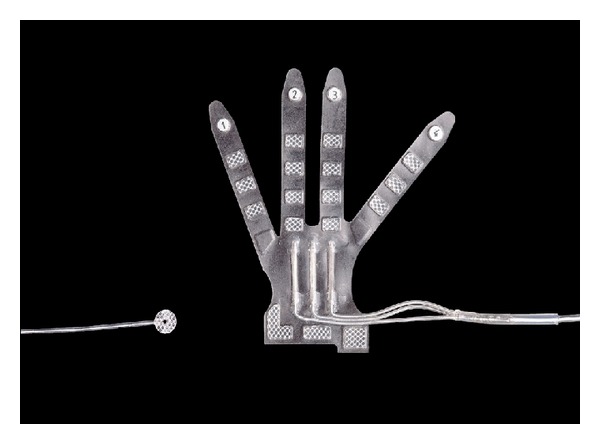
The carotid sinus electrodes of Rheos system (on the right) and Barostim *neo* (on the left). Figure permission was granted by CVRx, Inc.

The second-generation device (Barostim *neo*) has recently become available. It has received CE marking for use in resistant hypertension and continues to be studied in clinical trials. As detailed in Figure 1, the newest device consists of an IPG and only one carotid sinus electrode when compared to Rheos system. The IPG in Barostim *neo* provides extended battery longevity in a smaller size (see Figure 2). Furthermore, the programming system is by wireless telemetry to simplify connection to the device and modulation of electrical settings. As evident from Figure 3 the new electrode is substantially reduced in size and requires less power to provide the same benefit. The electrode is placed unilaterally, typically on the right carotid sinus via a small skin incision (2.5–5 cm). In case of a contraindication for right-sided implantation (significant carotid atherosclerosis, carotid bifurcation above the level of mandible), the electrode will be placed on the left carotid sinus. Generally, Barostim *neo* system is intended to deliver the same effects for hypertension treatment but reduces risks and duration of the surgical implantation and hospitalization.

## 4. Rheos System Human Studies

### 4.1. BRASS and DEBuT-HT

In 2003 the Baroreflex Activation System Study (BRASS), the first human proof-of-principle trial with the Rheos system, was performed. Acute voltage-dependent BP drop was observed in 11 normotensive patients undergoing an elective endarterectomy, which averaged 18 mmHg for systolic BP and 8 mmHg for diastolic BP [31]. This served as the basis for the phase II, multicenter, nonrandomized Device-Based Therapy of Hypertension Trial (DEBuT-HT) in 45 patients with drug-resistant hypertension. The participants showed a mean BP reduction of 33/22 mmHg after 2 years of follow-up [1]. The safety profile was acceptable, with in total 8 subjects who experienced a procedure- or device-related serious adverse event (SAE) [1].

### 4.2. Pivotal Trial

The Rheos Pivotal Trial was a randomized, double-blind, phase III trial. It was designed to assess the safety and efficacy of Rheos system [2]. Patients enrolled in this study were all experiencing resistant hypertension despite optimal and adherent antihypertensive therapy. A total of 265 patients in 49 centres were randomized in a 2 : 1 fashion and implanted with Rheos system. Group A consisted of patients who received BAT one month after implantation of the device (immediate BAT). Group B started BAT after month 6 of randomization (deferred BAT). 

In the primary efficacy endpoint Group A was compared to Group B for the proportion of subjects that achieved at least a 10 mmHg drop after 6 months of follow-up. The analysis showed 54% responders in Group A and 46% responders in Group B (**P* = 0.97* with 20% superiority margin). Furthermore, 88% of the responders in Group A maintained their response after 12 months of BAT. Mean decrease in systolic BP after 6 months was 16 ± 29 mmHg for Group A and 9 ± 29 mmHg for Group B (**P* = 0.08*). The proportion of subjects that achieved a systolic BP ≤140 mmHg at 6 months was significantly higher in Group A than in Group B (**P* = 0.005*). Both groups achieved a percentage of over 50% at month 12, at which point Group B already received BAT for 6 months (**P* = 0.70*) [2].

The safety analyses demonstrated an event-free rate of 74.8% for procedure safety. The most common procedure-related events were transient or permanent nerve injury. BAT as a therapy showed to be safe with 40% reduction in rate of hypertensive events in Group A. The device safety yielded an event-free rate of 87.2% after 12 months [2].

**Figure 4:**
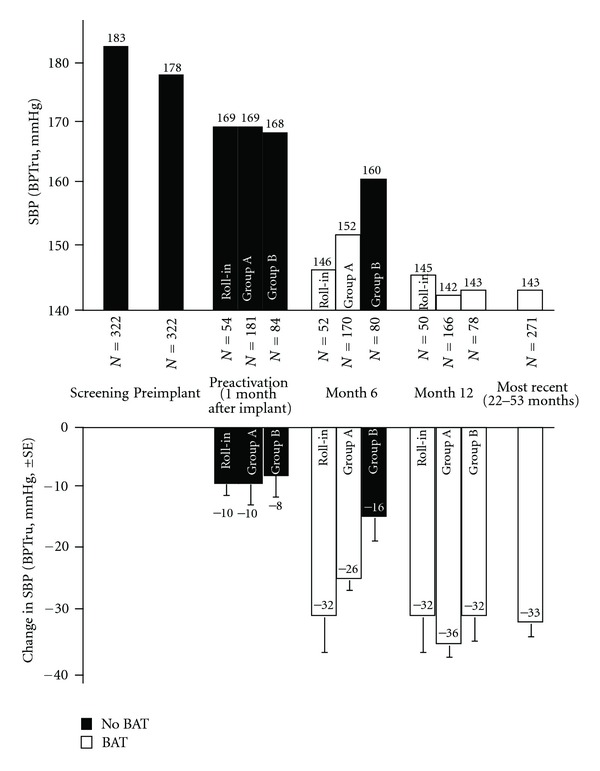
Systolic blood pressure values from screening, randomized phase, and long-term follow-up of subjects participating in Pivotal Trial. SBP : systolic blood pressure; BPTru : blood pressure measuring device; SE : standard error (Used from Bakris et al. [26]).

Although the trial did not meet all the prespecified endpoints, the overall weight of the provided evidence suggests that BAT can safely reduce BP over the long term. Recently published data showed significant BP reduction in long-term BAT [26].  Figure 4 illustrates a systolic BP drop of >30 mmHg by month 12 compared to preimplant systolic BP in the participants of Rheos Pivotal Trial. Nonetheless further studies are necessary to provide more insight in the benefits of BAT.

## 5. Toward Unilateral Baropacing

The Rheos system provides the ability to optimize and individualize the programming of the device for each patient. Although the electrodes of Rheos system were implanted bilaterally, the majority of participants were programmed unilaterally in the end [32]. Out of the 322 patients (including roll-in patients) enrolled in the pivotal trial 77% had a unilateral stimulation. Subjects with unilateral BAT showed a systolic BP reduction of 32 ± 3 mmHg and 31 ± 4 mmHg for right- and left-sided programming, respectively, after 6 months of BAT. This was comparable to patients who had bilateral BAT (21 ± 4 mmHg decrease in BP). These results suggest that it is not necessary to activate both left and right carotid baroreflex pathways to achieve maximum decrease in BPs. However, these data do not clearly suggest if there is a preferred side on which carotid sinus side should be stimulated.

Previous attempts to evaluate the individual effect of single left- or right-sided carotid baroreceptor activation did also not deliver conclusive results in humans. The idea of functional asymmetry and side dominance in the function of the carotid baroreflexes seems plausible. This may be due to right/left differences in cardiac innervation and projections of baroreceptor afferents to CNS [33]. Williamson et al. found left-sided dominance for MSNA by direct measurements from right peroneal nerve during unilateral sustained neck pressure by a neck collar device in 10 healthy volunteers [34]. In contrast, Furlan et al. showed no functional asymmetry in sympathetic discharge in response to unilateral neck suction in 12 healthy subjects [35]. Regarding the carotid-cardiac baroreflexes Williamson and Raven suggested that right and left cardiac reflexes are quantitatively similar [36], while right-sided baroreceptor activation was found more effective in modulating R-R interval by Tafil-Klawe et al. and Furlan et al. [33, 35]. Although animal data reported greater effects on heart rate with right-sided activation [37], comparisons of bilateral and unilateral baroreceptor activation and their effect on heart interval and peripheral resistance in humans provided conflicting findings [35, 36]. 

The development of the unilateral baropacers is certainly supported by the present Pivotal data. However, previous research did not provide enough basis to favor a specific carotid sinus side over the other. Therefore, further work is needed to determine what carotid sinus side should ideally be activated and to what amount interindividual variability is present in side dominance. Studies conducted with Barostim *neo* will probably add knowledge to the physiologic differences of unilateral carotid baroreflex function, as patients who lack adequate BP response to right-sided BAT have the opportunity to get an additional carotid sinus electrode implanted on the left side.

## 6. Barostim *neo* Human Studies

### 6.1. XR-1 Verification Study

The first human trial with the second generation of carotid baroreflex activators is the XR-1 Verification Study, which is currently in progress. The purpose is to assess the safety and efficacy of the Barostim *neo* system in patients with drug-resistant hypertension. Forty subjects are included in this nonrandomized, open-label trial at up to 15 clinical sites in Europe and Canada. All participants will be implanted with Barostim *neo* and therapy is started 2 weeks after implantation of the device. Preliminary data reported by Hasenfuss et al. [38] at the European Society of Cardiology meeting (ESC 2011) showed that systolic BP decreased by 28.7 mmHg in 12 patients after 3 months of continuous unilateral right-sided BAT. This is comparable to results obtained by the Rheos system. The safety profile of Barostim *neo* in 32 participants was substantially improved when compared to the first generation device. In the 30-day postprocedural safety only 3 complications occurred (pocket hematoma, self-inflicted wound complication, and device repositioning due to IPG discomfort). One system-related complication was reported, consisting of pain near the IPG. Final results of this study are still pending, but it seems that the new configurations of the second-generation device improved the safety profile of BAT [39].

## 7. BAT in Heart Failure

The mechanism of action of baroreceptors may make BAT a therapeutic tool for a wide range of cardiovascular diseases including heart failure. Beneficial effects of BAT on cardiac parameters have been observed in resistant-hypertensive patients treated with Rheos system. Data from the DEBuT-HT reported improvements in functional capacity. Kroon et al. [40] found an increase in 6-minute hall walk after 12 months of BAT. Improvements in cardiac structure were also observed by echocardiographic analyses [40, 41]. In a substudy of DEBuT-HT, Bisognano et al. presented a significant left ventricular mass index (LVMI) decrease from 138.9 ± 6.0 to 114.3 ± 3.9 g/m^2^ and a significant increase in median Left Ventricular Ejection Fraction (LVEF) from 65% to 67% after 12 months of continuous therapy [42]. Furthermore, BAT reduced left atrial dimension, left ventricular wall thickness, and mitral A-wave velocity. Cardiac data from pivotal trial also showed positive results in a substudy which included 46 patients. LVMI decreased from 117.7 ± 4.3 to 99.9 ± 3.0 g/m^2.7^ one year after activation [43]. Left ventricular mass decreased from 260.2 ± 11.9 to 222.9 ± 6.9 g in the same time period. These changes probably contribute to the improvement in diastolic function and provide an attractive strategy for the treatment of heart failure with preserved ejection fraction (HFpEF). A recent paper of Georgakopoulos et al. presents a clear overview of evidence suggesting BAT as a potential therapy for HFpEF [44]. 

Although current data demonstrated the effects of BAT only in hypertensive patients with various degrees of HFpEF, BAT may also be beneficial in patients with reduced EF (HFrEF) as these two share pathophysiologically much in common. A feasibility study is already ongoing to assess the potential benefit of Barostim *neo* BAT in patients with advanced heart failure. Main inclusion criteria are age between 21 and 80 years and symptomatic heart failure despite optimal, stable medical therapy for at least 4 weeks. Furthermore, a phase III XR-1-randomized heart failure study in patients with ischemic cardiomyopathy and reduced LVEF is going to start in the near future. Up to 300 subjects will be enrolled at about 30 clinical centres in Europe and Canada. Participants will be randomized in a 1 : 1 fashion to receive BAT on top of standard medical therapy or to receive optimal medical therapy alone. The primary efficacy endpoint will be to determine the change in left ventricular ejection fraction in subjects who receive BAT compared to standard medical care after 6 months of follow-up. 

## 8. Conclusions and Future Perspectives

The great necessity to alternative and effective BP reducing strategies created movement towards device-based therapies. BAT showed to be safe and effective for the treatment of drug-resistant hypertension. In addition, this approach seems to have potential in patients with heart failure and further efforts are being undertaken to evaluate the applicability in other cardiovascular diseases. However, various key issues remain to be identified in the field of carotid baropacing. The device is still undergoing progressive technical development to improve safety and increase the benefit and comfort for the user. Furthermore, uncertainties about the exact working mechanism and selection of optimal candidates for this therapy need to be explored. Future studies on BAT need to provide insight into appropriate patient selection and best device settings (unilateral versus bilateral activation and side-dominance) and expand the knowledge about the (patho) physiology and function of carotid baroreceptors in BP regulation.
